# With Our Editors

**Published:** 1907-04-15

**Authors:** 


### WITH OUR EDITORS.

**EXTRACTING FOR REGULATING.** Extraction for the so-called correction or irregularity is a snare and delusion. It has done far more harm than good in the past, and will continue to do so as long as employed. We do not include such work as extraction of supernumerary teeth in this category, but we do include all other kinds, especially extraction to "make room" for teeth. Such extraction invariably lessens the room instead of creating more. The dental arch collapses still more than its original condition, and the remaining teeth are worse crowded than before. This may not be apparent at first, but may be easily proved. Heaven knows there is altogether too much proof of it already existing, due to the efforts of well-meaning but much-mistaken doctors and dentists.

Extraction destroys the correct occlusion of the teeth, the fact on which permanency of form of the dental arch depends. There is no time in life but what the teeth will move from their positions and change the form of the dental arch if said arch is not rigidly maintained intact. The dental arch follows the same laws of mechanics as any other arch; if one or more of the stones be lost from over a vaulted doorway or window, that arch will collapse and there is no getting away from the fact. Extraction performs exactly the same office for the dental arch, and slowly but surely collapse of the dental structure follows. Nature does her best to correct the difficulty, and patches up the best occlusion possible from the wreck, but it is never a good one and often not even a serviceable one.

Right here somebody always rises to say, "But what if the occlusion is not quite perfect; I have extracted in many cases, and the teeth finally shifted around till my patient looked better, and the occlusion was good enough for all practical purposes." This statement *sounds* well enough, but Mr. Man, have you ever *really* investigated the conditions in one of these cases? Have you made models of the case year after year and noticed the change of form of the arch, and watched the gradual drift of the teeth about, and studied to see how some received practically all the wear and others none, and then noted how first one tooth and then another was lost years before its time? Have you mounted models of the case in an anatomical articulator (not an old barn-door hinge) and tested the occlusion, not just the open-and-shut motion, but the incising and lateral motions as well, and noted that the shearing action was all but lost where your extraction occurred; that often the teeth on a whole side were not in contact at all upon the slightest sidewise motion, and that your seeming good occlusion was in reality no occlusion at all; all this and plenty more besides? No, my dear boy, you have not made these tests or you would now be against extraction with all your might, for you would see its evil effects beyond mistake, and you would KNOW that extraction was wrong the same as the rest of us have learned from these very same things and many more like them.

No one need doubt in the matter of extraction, nor take any one's word in the case, for anyone who cares may easily test it himself, and that, too, without doing harm. Mount a set of *good* models of any case where extraction was occurred in an anatomical articulator and begin. Don't use the common articulator, for it is absolutely valueless in studying occlusion—or for any other purpose, for that matter. The mere striking together of the upper and the lower teeth does not constitute occlusion. The operation of mastication involves the gliding and sliding of all the teeth over each other during all motions of the jaw, and occlusion includes every variety of contact possible between the upper and lower teeth, both while in motion and while at rest. It takes a real articulator to represent this; the fake variety doesn't fill the bill.

It will be found that though there may be apparently good contact between uppers and lowers when in a state of rest, the moment the lower jaw is moved laterally there is failure to occlude properly in some particular, and usually in many particulars. And it is the proper interlocking and shearing during the *lateral motion* that holds the teeth firmly to their places, as well as performs the major part of mastication, two of the important facts connected with occlusion.

The experiment may be tried easily of extracting a tooth or two and then rearranging the teeth in a new occlusion. Mount up a set of models, more or less irregularly, but containing all the teeth, then saw off and lay aside a plaster tooth, say a first bicuspid, as is often sacrificed in the vain effort to correct through extraction. Then saw off a few of the others, as many as thought necessary, and wax them fast in new positions, moving them only such amounts and giving them such positions as can reasonably be expected through regulating. It will be found that it is a hard job to make any arrangement whatever whereby even a semblance to occlusion is gained, and utterly impossible to make an occlusion that performs any service upon the lateral motion. Such an occlusion would not hold the teeth in place, and such a case would not stay corrected when done. The experiment may be varied at will, leaving out first one tooth and then another. or "extracting" one from both upper and lower arches, or any other combination that is usually tried, but each and all will fail when the test of lateral motion is applied, and each would fail to be permanent if performed in actual practice.

There is neither mystery nor theory in the doctrine that extraction in orthodontia is wrong. Anyone may understand it, and demonstrate it for himself. Its bad results have been demonstrated practically—alas! time and time again, and, it must be regretfully stated, it will be practiced yet other times by those who ought to know better. In years gone by they believed the world was flat—were certain of it—till one fine day a man completed a voyage entirely around it. All the previous philosophy and teaching concerning the world's flatness went out of date in an instant, yet they could not give up the good old beliefs for a long time after. The question of extraction in orthodontia is exactly similar; it has been proved wrong; don't be one of those who still believes the earth is flat.—William J. Brady, D. D. S. in *Western Journal.*

**DRAWING THE LINE.** There has ever been a tendency on the part of a few members, both of the medical and dental professions, to draw a line of demarkation between the science and art of the same. To regard the scientist as in some way superior to the artisan in the same calling. Of course, such a distinction is an absurdity, and yet it has had its influence on the young men who have entered on the practice of dentistry in the last decade. Without a degree of scientific knowledge, manual skill becomes nearly useless, and without manual training theoretical skill is wasted. It is like drawing a line between the importance of the respiration and the circulation, or saying that operative and prosthetic dentistry can be made separate sciences.

The effect of constantly extolling the beauties of original research and investigation has been to give many beginners the idea that the mere manipulative procedures incident to their profession are of minor importance. And especially in certain lines of prosthetic dentistry it has grown into a custom to relegate the work to a laboratory where it is turned out at wholesale prices, and by machine-made patterns. This idea is all wrong and is responsible for much of the cheap and shoddy dental work that is in evidence today.

There is no more credit in inserting a beautiful inlay than in inserting a beautiful vulcanite plate—provided each was indicated in its respective place. There is no more glory due the man who discovers a new and pestiferous germ than the one who discovers an improved method of taking an impression or making a crown.

There has been a distinct lowering in the quality of the plate work done throughout the country in the last twenty years. Of course, this is partly due to the improvements in bridge work. But if a plate is indicated at all, the best possible plate is indicated. The conscientious surgeon lances a bol, with as much care as he cuts off a leg. The conscientious dentist, in his sphere, should do no less.

In conversation, not long go, with a dentist, practicing in an interior Kansas town, the writer was extolling porcelain work in general, inlays, crowns, continuous gum plates, etc. "Oh, yes," remarked the listener, "that's all very well for you city fellows, but I never have a call for anything of that kind out where I live." Of course not. No one is going to call for something of which he never heard. As a matter of fact, the people take, in the way of professional service just what is offered them. The fact that one style of operation, on account of material used, may be somewhat more expensive than another will have very little bearing. The American people are celebrated for wanting the best of everything. They wear the best clothes, eat the best food, live in the best houses and have the best time of any people on earth. The dentist who fails to give them the best available in his line is derelict in his duty and blind to his opportunities.—Dr. F. G. Wortley, in *Western Journal.*

**BETTER SOCIETY ORGANIZATION.** The article on this subject which appeared in the December *Digest* has attracted considerable attention throughout the country, as evidenced by letters received, and we believe the time is close at hand when there will be a general reorganization of our state societies. In fact this time seems to have arrived, for Iowa, Missouri, Wisconsin, Michigan and Southern California have already undertaken the work on plans similar to those carried out in Illinois, and the secretary of the Illinois State Dental Society has received letters from Vermont, Pennsylvania, Ohio, North Carolina, Indiana, Kentucky, Mississippi, Kansas, Nebraska, Minnesota, Colorado, Texas, Oklahoma, Florida and other states which have the adoption of such a plan under consideration.

It is probable that the total membership of all of out state organizations is less than ten per cent of the practitioners of dentistry in the United States. This does not mean that only ten per cent of our dentists are society men, as many belong to local and district organizations without being members of the state societies. It is probable that fully three-fourths of the dentists of the country are without society connection. These men must rely principally on our dental publications for new ideas and methods. Society work has , therefore, no influence with these men, except as they see it reported in the journals. The fact that three-fourths of the profession receive little benefit from society work is a bad enough condition, but the fact that this same three-fourths are doing little or nothing as their share towards the advancement of the profession is much more deplorable.

We are very much of the opinion that it is by no means entirely the fault of the men outside of our societies that they are not members. It is rather the failure of the society men to extend the proper invitation. In Illinois over twelve hundred new members have been taken in and the society has now maintained its large membership for the third year, indicating that the new men have largely joined to stay and are doing their part in the work of advancement.

The component society plan of organization gives each man an opportunity to do something, and get well acquainted with fellow practitioners of the community. It is rapidly becoming apparant in Illinois that the establishment of goodfellowship is and should be the basis of all effective society work. Whenever a feeling of goodfellowship has been established among the dentists of any section, those men treat each other and each other's patients well and are willing to give and take in all things. When the feeling is right among the men of a component society , all other things are easy; when the feeling is not right, it is practically impossible to accomplish anything.—J. N. Crouse in February *Digest.*

**YOUR VACATION.** With springtime at hand it means to you, brother dentist, a great deal of hard nerve-racking work, but at the same time you should think of your *vacation.* If any professional man under the sun needs a rest it is the dentist, who is confined to his office practically the year around, for at least six days in the week, and we find a great many who spend seven days, with long hours at that. A man might be of healthy robust physique, eating three square meals a day and enjoining the merciful bounties of this "vale of tears" to his utmost capacity, but take and place him in an eight by eight operating room and have him thus with his patients to take care of for the year around and notice the change, for change there must come, even though he still appears outwardly the same, he eats less, and with the constant strain on his mind, his constitution becomes run down and later, with this ceaseless grind working on him, that individual will find himself a weakened, nervous, irritable dyspeptic.

"Can't find time for a vacation," you say? Well, *just take time,* for you'll never make a better investment, one which will give you returns almost beyond estimation. You're just the man that needs a rest. Go on a fishing trip, where all you have to think of is fresh air and water, and let the practice take care of itself. A man's health is worth more than any amount of wealth. Don't be a slave. If you don't like fishing take a trip of some kind or do something which will take you away from the confines of your office and practice.

How much better you can do when you've taken a good vacation. Your work is not a drudge like before, but comes and goes in the most pleasing manner and everything seems to have a brighter aspect. Your hand, which was nervous and uncertain before, is steady, and you feel as you take a deep inspiration and rise to your fullest height,that you could lick "the best man that ever entered a ring."

Again , a man owes his best and most earnest efforts to his professional calling, but still he owes a great deal to himself and therefore let's figure on that *vacation*, something extrinsic to the usual routine of work.—*March Odontoblast.*

